# Effect of changes in inspired oxygen fraction on oxygen delivery during cardiac surgery: a substudy of the CARROT trial

**DOI:** 10.1038/s41598-021-97555-2

**Published:** 2021-09-09

**Authors:** Karam Nam, Hye-Bin Kim, Young-Lan Kwak, Young Hyun Jeong, Jae-Woo Ju, Jinyoung Bae, Seohee Lee, Youn Joung Cho, Jae-Kwang Shim, Yunseok Jeon

**Affiliations:** 1 Department of Anesthesiology and Pain Medicine, Seoul National University Hospital, Seoul National University College of Medicine, Seoul, 03080 Korea; 2 Department of Anesthesiology and Pain Medicine, Anesthesia and Pain Research Institute, Yonsei University College of Medicine, Seoul, 03722 Korea

**Keywords:** Physiology, Cardiology

## Abstract

When hemoglobin (Hb) is fully saturated with oxygen, the additional gain in oxygen delivery (DO_2_) achieved by increasing the fraction of inspired oxygen (FiO_2_) is often considered clinically insignificant. In this study, we evaluated the change in DO_2_, interrogated by mixed venous oxygen saturation (SvO_2_), in response to a change in FiO_2_ of 0.5 during cardiac surgery. When patients were hemodynamically stable, FiO_2_ was alternated between 0.5 and 1.0 in on-pump cardiac surgery patients (pilot study), and between 0.3 and 0.8 in off-pump coronary artery bypass grafting patients (substudy of the CARROT trial). After the patient had stabilized, a blood gas analysis was performed to measure SvO_2_. The observed change in SvO_2_ (ΔSvO_2_) was compared to the expected ΔSvO_2_ calculated using Fick’s equation. A total 106 changes in FiO_2_ (two changes per patient; total 53 patients; on-pump, n = 36; off-pump, n = 17) were finally analyzed. While Hb saturation remained near 100% (on-pump, 100%; off-pump, mean [SD] = 98.1% [1.5] when FiO_2_ was 0.3 and 99.9% [0.2] when FiO_2_ was 0.8), SvO_2_ changed significantly as FiO_2_ was changed (the first and second changes in on-pump, 7.7%p [3.8] and 7.6%p [3.5], respectively; off-pump, 7.9%p [4.9] and 6.2%p [3.9]; all P < 0.001). As a total, regardless of the surgery type, the observed ΔSvO_2_ after the FiO_2_ change of 0.5 was ≥ 5%p in 82 (77.4%) changes and ≥ 10%p in 31 (29.2%) changes (mean [SD], 7.5%p [3.9]). Hb concentration was not correlated with the observed ΔSvO_2_ (the first changes, *r* =  − 0.06, P = 0.677; the second changes, *r* =  − 0.21, P = 0.138). The mean (SD) residual ΔSvO_2_ (observed − expected ΔSvO_2_) was 0%p (4). Residual ΔSvO_2_ was more than 5%p in 14 (13.2%) changes and exceeded 10%p in 2 (1.9%) changes. Residual ΔSvO_2_ was greater in patients with chronic kidney disease than in those without (median [IQR], 5%p [0 to 7] vs. 0%p [− 3 to 2]; P = 0.049). DO_2_, interrogated by SvO_2_, may increase to a clinically significant degree as FiO_2_ is increased during cardiac surgery, and the increase of SvO_2_ is not related to Hb concentration. SvO_2_ increases more than expected in patients with chronic kidney disease. Increasing FiO_2_ can be used to increase DO_2_ during cardiac surgery.

## Introduction

The ultimate goal of hemodynamic management is to optimize oxygen transport and maintain adequate tissue oxygenation. Shoemaker et al. demonstrated in their early study that reduced oxygen transport was a predictor of death after major surgery for life-threatening shock^1^. The concept of oxygen transport optimization evolved following that study, and has become an important component of goal-directed hemodynamic management^2^.

Convective oxygen transport describes oxygen delivery (DO_2_) to peripheral tissues and organs via the circulation system, which can be managed by monitoring mixed venous oxygen saturation (SvO_2_)^3,4^. DO_2_ is a product of cardiac output (CO) and arterial oxygen content (CaO_2_)^3,4^, and CaO_2_ is a function of hemoglobin (Hb), arterial oxygen saturation (SaO_2_), and arterial oxygen partial pressure (PaO_2_), described as follows^5^:

$${CaO}_{2}={(k}_{1}\times Hb\times {SaO}_{2})+{(k}_{2}\times {PaO}_{2})$$

where *k*_1_ (Hüfner’s constant) and *k*_2_ (Bunsen’s coefficient) are approximately 1.34 ml/g and 0.0034 ml/dl/mmHg, respectively. As can be inferred from this equation, the theoretical contribution of PaO_2_ to DO_2_ is negligible compared to the Hb concentration^3,6^. Consequently, it is a generally accepted idea that an increase in DO_2_ that can be achieved by increasing the fraction of inspired oxygen (FiO_2_) is minimal after Hb is saturated. This concept can lead physicians to overlook the importance of FiO_2_ adjustment in perioperative DO_2_ management.

Therefore, based on our clinical experience, we hypothesized that a significant increase in DO_2_ could be achieved by increasing FiO_2_ (and PaO_2_), even after Hb is fully saturated in cardiac surgery patients. To evaluate this hypothesis, we analyzed the effect of changing FiO_2_ on DO_2_ reflected as SvO_2_ in patients undergoing cardiac surgery.

## Results

**Figure 1:**
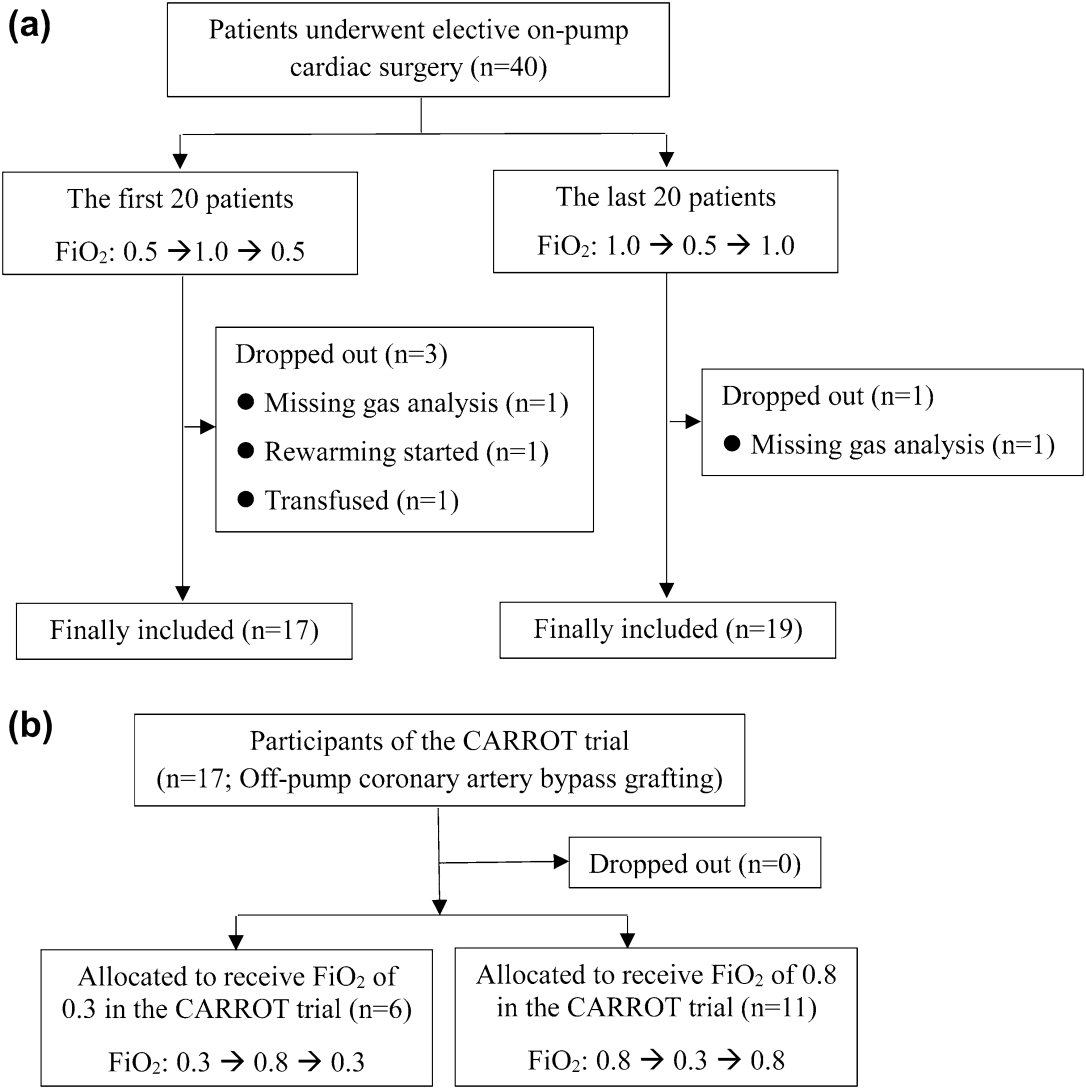
Study flow chart of (**a**) on-pump and (**b**) off-pump patients. FiO_2_, fraction of inspired oxygen.

The study flow chart is presented in Fig. 1. Among the on-pump cardiac surgery patients (n = 40) enrolled in protocol 1 (see the “Methods” section), four dropped out because the blood gas results were missing (n = 2), rewarming was started during the study (n = 1) or red blood cells were transfused during the study (n = 1) (Fig. Methods1a). None of the participants (n = 17) of the CARROT trial who underwent off-pump coronary artery bypass grafting (OPCAB) dropped out from protocol 2 (Fig. 1b; see the “” section). Missing values were omitted without data imputation. The remaining 53 patients (on-pump, n = 36; OPCAB, n = 17) were included in the final analysis (Fig. 1).

The patient characteristics are described in Table 1. The mean (SD) Hb concentration was 7.7 g/dl (1.3), and the mean nasopharyngeal temperature was 29.2 °C (1.5), in on-pump patients following protocol 1. The mean Hb concentration and the nasopharyngeal temperature were 11.5 g/dl (2.1) and 35.8 °C (0.6), respectively in OPCAB patients following protocol 2. The mean cardiopulmonary bypass (CPB) flow rate was 4.1 l/min (0.5) in on-pump patients, and the mean CO measured via a pulmonary artery catheter using the thermodilution method was 3.3 l/min (0.5) in OPCAB patients. The hemodynamic variables measured at T0–T2 throughout the study are presented in Supplementary Table S1 online.

**Table 1:** Demographics and baseline characteristics of the study population.

	On-pump (n = 36)	Off-pump (n = 17)
Age (years)	61.4 (12.4)	65.7 (7.6)
Female	18 (50.0%)	6 (35.3%)
Height (cm)	161.8 (10.9)	161.5 (10.2)
Weight (kg)	63.3 (14.4)	66.4 (8.5)
**Comorbidities**		
Hypertension	11 (30.6%)	12 (70.6%)
Diabetes	5 (13.9%)	11 (64.7%)
Chronic kidney disease	1 (2.8%)	5 (29.4%)
Cerebrovascular disease	5 (13.9%)	1 (5.9%)
Chronic obstructive lung disease	0 (0%)	0 (0%)
Infective endocarditis	1 (2.8%)	0 (0%)
Congestive heart failure	11 (30.6%)	2 (11.8%)
**Medication history**		
ACEi or ARB	9 (25.0%)	7 (41.2%)
Beta blockers	14 (38.9%)	7 (41.2%)
Calcium channel blockers	7 (19.4%)	5 (29.4%)
Diuretics	26 (72.2%)	5 (29.4%)
Statins	13 (36.1%)	23 (63.9%)
**Surgery type**		
Coronary artery bypass grafting	0 (0%)	17 (100%)
Valve	17 (47.2%)	NA
Thoracic aorta	2 (5.6%)	NA
Valve + Coronary	1 (2.8%)	NA
Valve + Thoracic aorta	6 (16.7%)	NA
Valve + Maze procedure	7 (19.4%)	NA
Miscellaneous	3 (8.3%)	NA
**Surgical profiles**		
Redo surgery	6 (16.7%)	0 (0%)
Surgery duration (min)	327 (71)	362 (44)
CPB duration (min)	163 (53)	NA
**Laboratory data**		
Ejection fraction (%)	57 (10)	53 (13)
Serum creatinine (mg/dl)	0.8 (0.2)	1.4 (1.9)
Glomerular filtration rate (ml/min/1.73 m^2^)	86 (18)	76 (26)
Hemoglobin (g/dl)*	7.7 (1.3)	11.5 (2.1)
Hemodynamic data*		
Core body temperature (°C)^†^	29.2 (1.5)	35.8 (0.6)
Mean blood pressure (mmHg)	62 (6)	75 (13)
Cardiac output (l/min)	4.1 (0.5)^‡^	3.3 (0.5)
Cardiac index (l/min/m^2^)	2.5 (0.2)^‡^	1.9 (0.2)

Data are presented as mean (SD) or number (%).

*ACEi* angiotensin-converting enzyme inhibitors, *ARB* angiotensin II receptor blockers, *CPB* cardiopulmonary bypass.

*Values measured at T0.

^†^Measured at the nasopharynx.

^‡^Based on the pump flow rate.

### Comparison of SvO_2_ levels measured at different FiO_2_ levels

**Figure 2:**
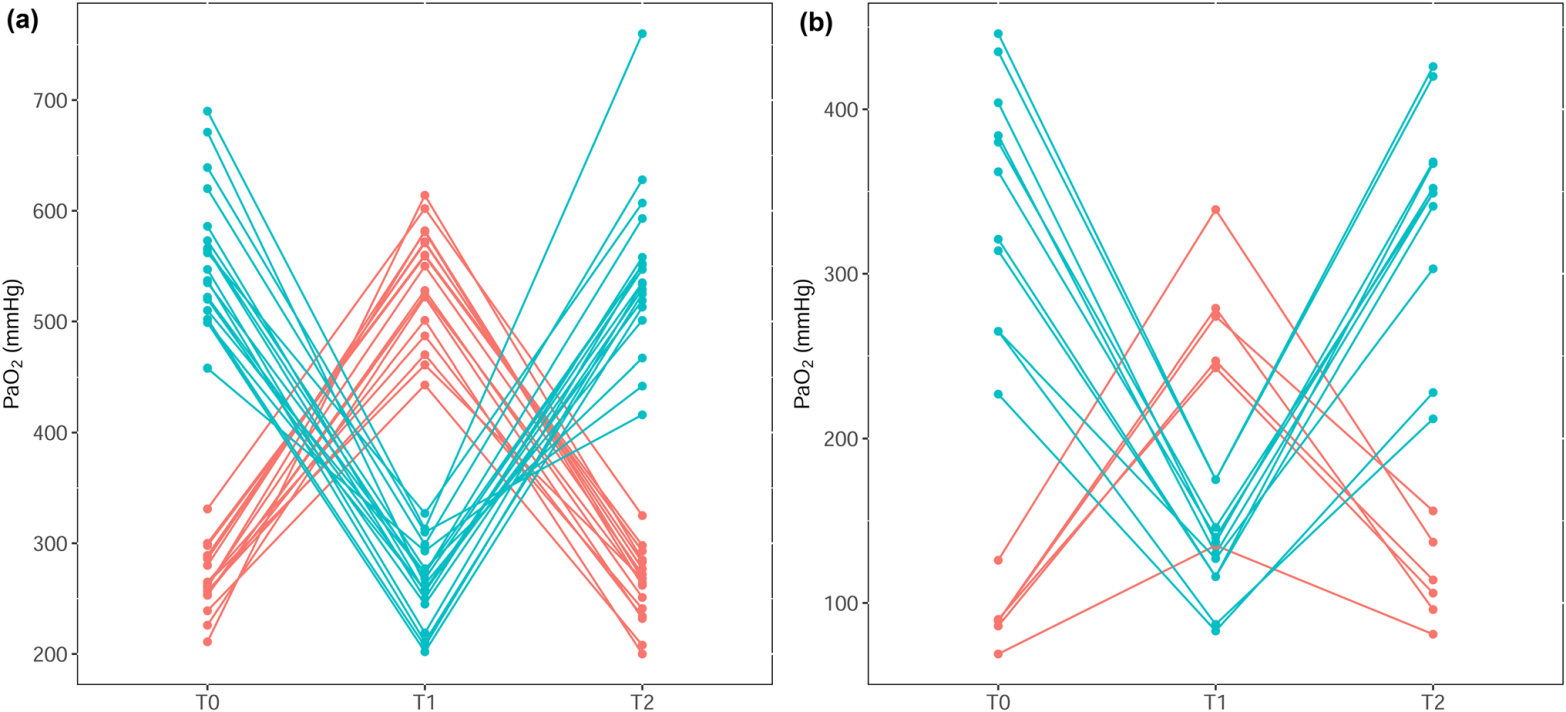
Change of PaO_2_ according to that of fraction of inspired oxygen in (**a**) on-pump and (**b**) off-pump patients. PaO_2_, arterial oxygen partial pressure. Created with R: A language and environment for statistical computing. R Foundation for Statistical Computing, Vienna, Austria; https://www.R-project.org/.

SaO_2_ remained relatively constant during both protocols. SaO_2_ was 100% in all on-pump cardiac surgery patients at every FiO_2_ level. In OPCAB patients, the mean (SD) SaO_2_ was 98.1% (1.5) when FiO_2_ was 0.3 and 99.9% (0.2) when FiO_2_ was 0.8. The pattern of PaO_2_ change in response to the change of FiO_2_ in every patient is shown in Fig. 2 and Supplementary Table S1 online.

**Figure 3:**
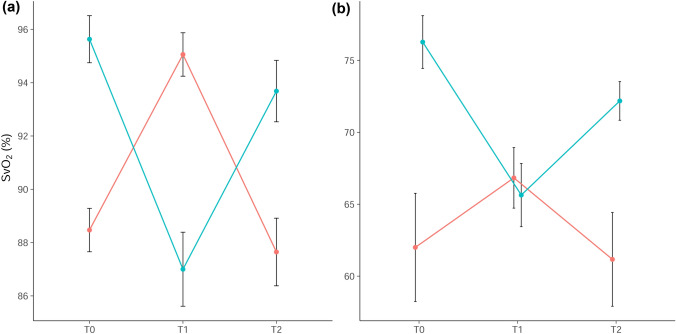
Change of SvO_2_ according to different FiO_2_ levels in (**a**) on-pump and (**b**) off-pump patients. SvO_2_, mixed venous oxygen saturation; FiO_2_, fraction of inspired oxygen. Created with R: A language and environment for statistical computing. R Foundation for Statistical Computing, Vienna, Austria; https://www.R-project.org/.

The changes in SvO_2_ throughout the study period are shown in Fig. 3. SvO_2_ changed significantly with the change of FiO_2_ (and PaO_2_) in on-pump cardiac surgery patients (mean [SD], T0–T1 7.7%p [3.8] and T1–T2 7.6%p [3.5]; both P < 0.001) and OPCAB patients (T0–T1 7.9%p [4.9] and T1–T2 6.2%p [3.9]; both P < 0.001). Regardless of the surgery type, 82 (77.4%) changes had an observed ΔSvO_2_ ≥ 5%p and 31 (29.2%) had an observed ΔSvO_2_ ≥ 10%p (mean [SD], 7.5%p [3.9]).

### ΔSvO_2_ according to Hb concentration

**Figure 4:**
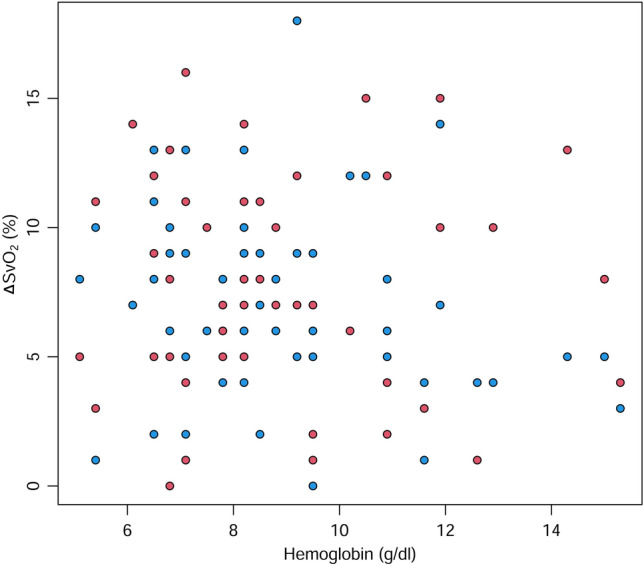
The relationship between ΔSvO_2_ and hemoglobin concentration. ΔSvO_2_, change of mixed venous oxygen saturation. Red dots, T0–T1; blue dots, T1–T2. Created with R: A language and environment for statistical computing. R Foundation for Statistical Computing, Vienna, Austria; https://www.R-project.org/.

The relationship between Hb concentration and ΔSvO_2_ is shown in Fig. 4. The Hb concentration (within the range 5.1–15.3 g/dl) was not correlated with the observed ΔSvO_2_ (T0–T1, *r* =  − 0.06, P = 0.677; T1–T2, *r* =  − 0.21, P = 0.138).

### Comparison of the observed and expected ΔSvO_2_

**Figure 5:**
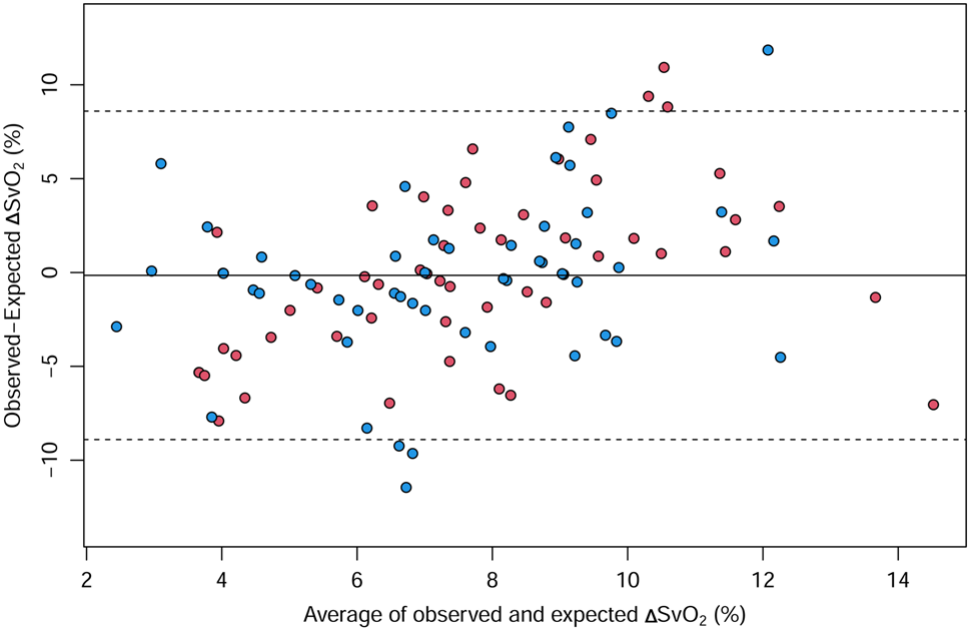
Bland–Altman plot for the observed versus the expected ΔSvO_2_. Dashed lines indicate the limits of agreement (the mean ± 1.96 × the standard deviation of the residual ΔSvO_2_). ΔSvO_2_, change of mixed venous oxygen saturation. Red points, T0–T1; blue points, T1–T2. Created with R: A language and environment for statistical computing. R Foundation for Statistical Computing, Vienna, Austria; https://www.R-project.org/.

The Bland–Altman plot for the observed and expected ΔSvO_2_ is presented in Fig. 5. Overall, SvO_2_ changed following a change in FiO_2_. The maximum residual ΔSvO_2_ (observed − expected ΔSvO_2_) was 12%p, and the mean (SD) residual ΔSvO_2_ was 0%p (4). Residual ΔSvO_2_ was more than 5%p in 14 (13.2%) changes. Residual ΔSvO_2_ exceeded 10%p in two changes.

### Exploratory analysis of factors associated with residual SvO_2_

**Figure 6:**
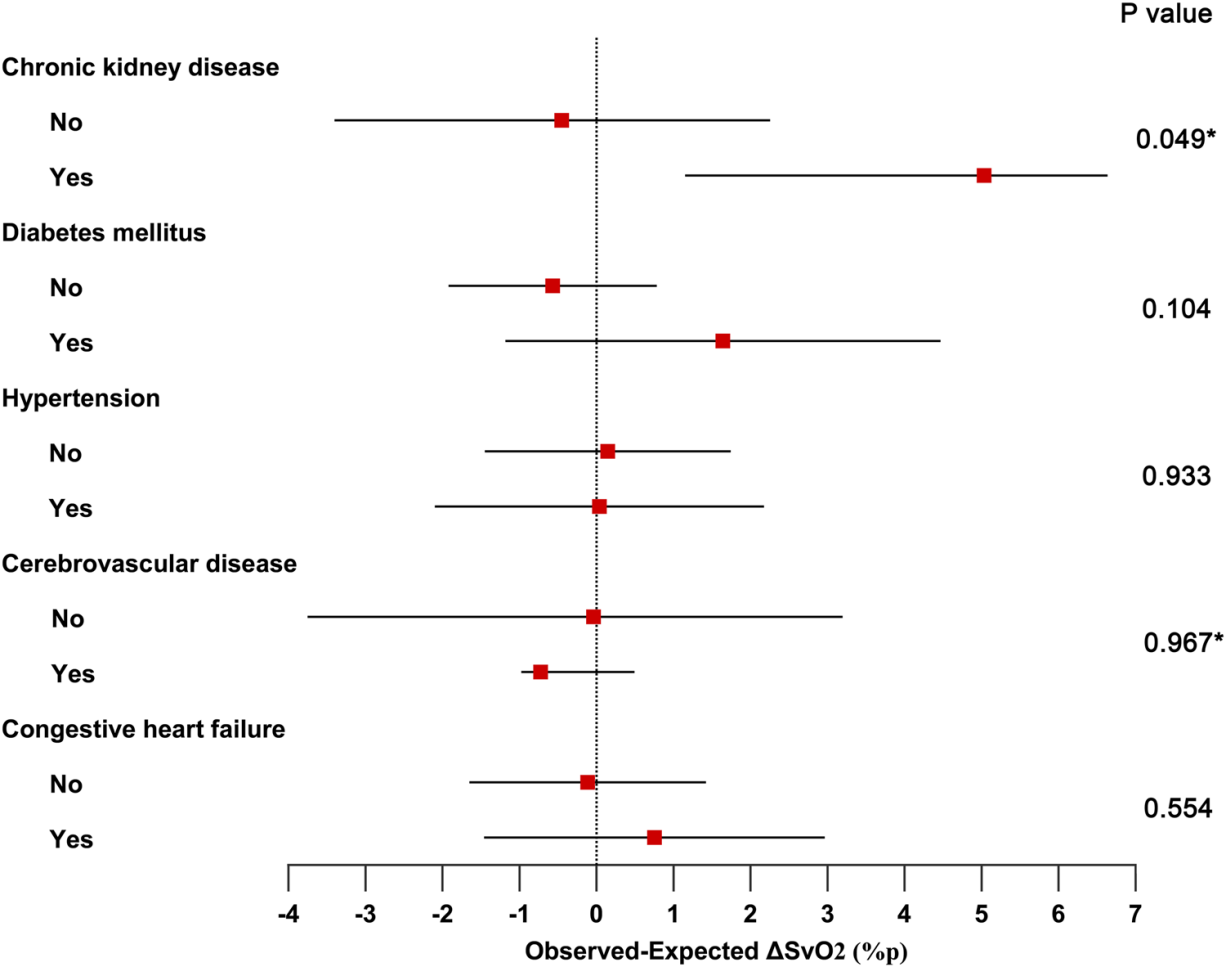
Comparison of the residual ΔSvO_2_ according to comorbidities. Asterisks refer to non-parametric results. Points indicate the mean or the median, lines 95% confidence interval or interquartile range. ΔSvO_2_, change of mixed venous oxygen saturation. R Foundation for Statistical Computing, Vienna, Austria; https://www.R-project.org/.

A forest plot summarizing residual ΔSvO_2_ according to preoperative comorbidities is presented in Fig. 6. Patients with chronic kidney disease (n = 6) had significantly greater residual ΔSvO_2_ than those without chronic kidney disease (n = 47) (median [IQR], 5%p [0 to 7] vs. 0%p [− 3 to 2]; P = 0.049) (Fig. 6). However, no significant difference was observed in residual ΔSvO_2_ between patients with and without diabetes (n = 16 and 37, respectively; mean [SD], 2%p [5] vs. − 1%p [4]; P = 0.104), or between those with and without hypertension (n = 23 and 30, respectively; 0%p [5] vs. 0%p [4]; P = 0.933). Residual ΔSvO_2_ also did not differ according to whether patients had cerebrovascular disease or not (n = 6 and 47, respectively; median [IQR], − 1%p [− 1 to 1] vs. 0%p [− 4 to 3]; P = 0.967), or whether they had congestive heart failure or not (n = 13 and 40, respectively; mean [SD], 1%p [4] vs. 0%p [5]; P = 0.544).

### The change of Hb equivalent that increases DO_2_ to the same extent as ΔFiO_2_ of 0.5

**Figure 7:**
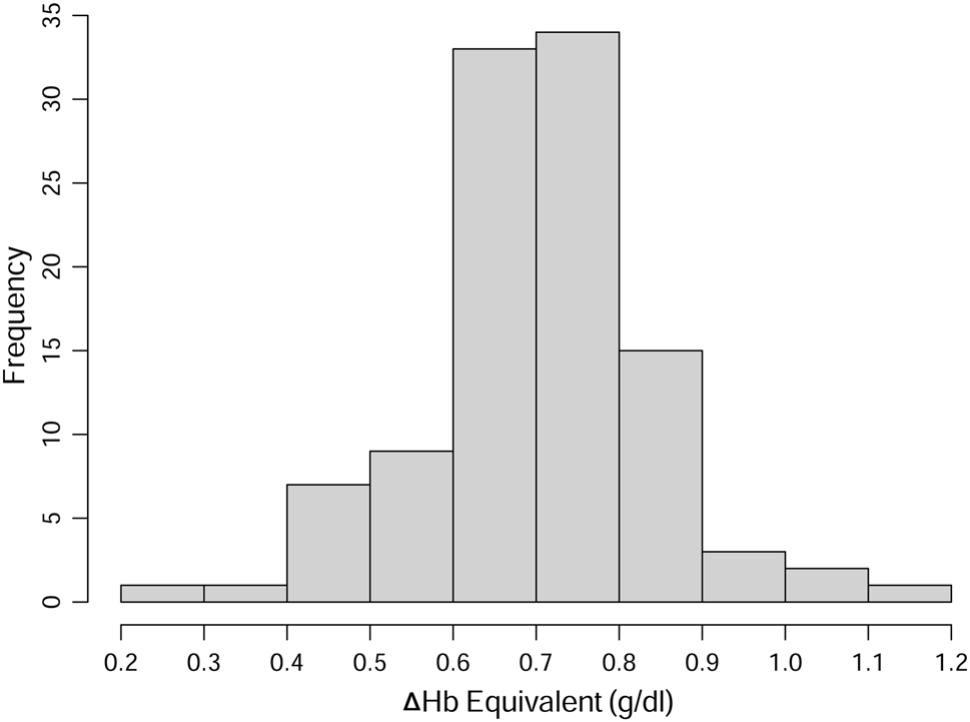
Distribution of the ΔHb equivalent that increases oxygen delivery to the same extent as ΔFiO_2_ of 0.5. ΔHb, change of hemoglobin concentration. Created with R: A language and environment for statistical computing. R Foundation for Statistical Computing, Vienna, Austria; https://www.R-project.org/.

The median (IQR) ΔHb equivalent that increases DO_2_ to the same extent as ΔFiO_2_ of 0.5 was 0.7 (0.6–0.8) g/dl. The maximum value was 1.1 g/dl. The distribution of the ΔHb equivalent values is presented as a histogram in Fig. 7. In more than 90% of changes with a change of FiO_2_, ΔFiO_2_ of 0.5 was equivalent to an ΔHb of more than 0.5 g/dl (97 changes, 91.5%), suggesting that use of a higher FiO_2_, at least temporarily, can achieve a similar effect as transfusion in terms of DO_2_.

## Discussion

In this study, SvO_2_ increased by ≥ 5%p in more than three-quarters of FiO_2_ changes where FiO_2_ was increased from 0.3 to 0.8 or 0.5 to 1.0 during cardiac surgery, and by ≥ 10%p in more than one-quarter of FiO_2_ changes. While Hb remained almost fully saturated, PaO_2_ changed remarkably as FiO_2_ was changed. There was no significant association between Hb concentration and ΔSvO_2_. These results indicate that DO_2_ can increase significantly following an increase in PaO_2_ induced by raising FiO_2_ during cardiac surgery. The median ΔHb equivalent to the FiO_2_ change of 0.5, in terms of its ability to increase DO_2_, was 0.7 g/dl. In addition, SvO_2_ tended to increase beyond the expected value that was calculated using the Fick’s equation, in patients with chronic kidney disease.

In most patients undergoing cardiac surgery, SaO_2_ is maintained at nearly 100% due to supplemental oxygen therapy, unless there is a significant shunt or pulmonary morbidity. In our study, SaO_2_ was 100% at every FiO_2_ level during on-pump cardiac surgery, and more than 98% and 99% at the FiO_2_ levels of 0.3 and 0.8, respectively, during OPCAB. In such a situation, it is generally expected that the contribution of PaO_2_ to DO_2_ will be much smaller than that of Hb-bound oxygen^3,6^; thus, manipulating FiO_2_ would have very little influence on SvO_2_ (or DO_2_)^5^. Therefore, clinicians may focus only on Hb concentration and transfusion when optimizing DO_2_.

Several studies have shown that perioperative DO_2_ management is associated with complications after cardiac surgery, such as neurologic injury^8–11^ and renal dysfunction^12–14^. However, previous studies mostly evaluated the effect of CO and Hb concentration rather than FiO_2_^8,9,13^. Hogue et al. reported that atrial fibrillation accompanied by low CO had a significant effect on the likelihood of postoperative stroke^8^. Bahrainwala et al. explained the link between reduced DO_2_ and postoperative stroke in terms of a decrease of Hb concentration alone^9^. Ranucci et al. also showed that severe hemodilution during CPB increases the risk of renal dysfunction, but emphasized that this can be attenuated by increasing DO_2_ with raising CO (pump flow)^13^.

Early studies by Clowes et al.^15^ and Shoemaker et al.^1^ revealed that survivors of peritonitis and shock have consistently higher DO_2_ and oxygen consumption (VO_2_) than those who died. Although our study showed that higher FiO_2_ significantly elevates DO_2_, this does not necessarily mean that the use of high FiO_2_ would improve clinical outcomes: there are several issues that need to be addressed. First, there is growing concern about the harmful effects of hyperoxia caused by high FiO_2_, although most previous clinical studies failed to demonstrate significantly poorer clinical outcomes due to hyperoxia or high FiO_2_^16,17^. Second, DO_2_ can increase in response to transfusion or intravascular volume expansion, but we do not know whether achieving the same level of SvO_2_ with different modalities results in an equivalent distribution of oxygen to the organs; the distribution of oxygen supply and demand differs among organs^18^. Weinrich et al. failed to find a correlation between surgical site oxygen saturation and central venous oxygen saturation in patients undergoing major non-cardiac surgery^19^. Similar findings have been reported in patients undergoing CPB cardiac surgery, where a significant difference between SvO_2_ and venous oxygen saturation measured at the brain or gut was demonstrated^20,21^. This heterogeneity not only exists at the global level, but also at the regional level within an organ^22^. However, these are poorly investigated topics, so further studies on are necessary. Currently, there is no firm consensus or established guidelines regarding the optimal oxygen therapy for patients undergoing cardiac surgery, and the present study did not answer this question. We are conducting a multicenter, cluster-randomized trial (the CARROT trial; Clinicaltrials.gov, NCT03945565) to compare the effects of different levels of intraoperative FiO_2_ (0.3 vs. 0.8) on clinical outcomes after OPCAB, including the length of postoperative hospital stay and major organ injuries.

Recent large-scale randomized trials, such as the TRICS III^23^ and the TITRe2^24^, failed to demonstrate a difference between restrictive and liberal transfusion strategies in terms of composite adverse outcomes after cardiac surgery. In these trials, only Hb concentration was tested as a trigger for red blood cell transfusion^23–25^. However, from the present study, and our previous study^26^ it can be inferred that there may be unknown interactions or confounders that make interpretation of the effect of transfusion on outcomes more complex. Although establishing the Hb threshold is currently the highest priority for transfusion and DO_2_ optimization, oxygen therapy and plasma dissolved oxygen should also be considered.

The present study had several limitations. First, only a small number of patients were included without an a priori sample size calculation. Furthermore, two heterogeneous groups of patients (on- and off-pump cardiac surgery patients) with different hemodynamic statuses and comorbidities were enrolled. The levels of FiO_2_ also differed between the study protocols. Moreover, we only assessed the immediate effect of a change in FiO_2_ on SvO_2_, and did not evaluate whether increasing SvO_2_ using a higher FiO_2_ ameliorates oxygenation of vital organs (which would improve clinical outcomes). We expect that the CARROT trial will answer these questions. Second, several (important) variables, such as CO, Hb concentration, and VO_2_, were assumed to be constant during the FiO_2_ changes for this analysis, which was inevitable for the calculation of expected ΔSvO_2_. To minimize the influence of these values, the both protocols were conducted when it was considered the most hemodynamically stable with the least surgical manipulation (see the “Methods” section). Obviously, changes in these variables were minimal during the study period (Supplementary Table S1 online), but may have affected the observed and expected ΔSvO_2_ values to a certain extent. Third, we only included cardiac surgery patients in this study. Thus, our results may not be applicable to patients in other settings, such as non-cardiac surgery patients and non-surgical, critically ill patients. Fourth, this study did not uncover the mechanism, or assess the clinical impact, of the phenomenon whereby a change in SvO_2_ caused by a change in FiO_2_ was larger than expected in patients with chronic kidney disease.

In conclusion, DO_2_, interrogated by SvO_2_, may be significantly elevated by increasing FiO_2_ during cardiac surgery. Increasing FiO_2_ may be considered when an increase in DO_2_ is necessary during cardiac surgery. However, considering the potential risk of hyperoxia, further studies evaluating the clinical effect of this practice are necessary.

## Methods

### Study population

This study was comprised of on-pump cardiac surgery and OPCAB parts. The part involving patients undergoing CPB cardiac surgery was a pilot study, which was approved by the Institutional Review Board of Seoul National University Hospital (IRB no., 1909-145-1067) and registered at ClinicalTrials.gov (NCT04144205). The other part, for patients undergoing OPCAB, was a substudy of the CARROT trial (IRB no., 1902-021-1008; ClinicalTrials.gov, NCT03945565).

The present study was performed in compliance with the guidelines for Good Clinical Practice and the Declaration of Helsinki. All participants recruited to this study provided written informed consent.

### Protocol 1: on-pump cardiac surgery

This part of the study was a pilot study for a future multicenter, randomized trial. Forty patients who presented for elective CBP cardiac surgery between November 4, 2019 and February 11, 2020 were enrolled in this study. There was no a priori sample size calculation. The exclusion criteria were preoperative supplemental oxygen at a dose equivalent to FiO_2_ of > 0.5, symptomatic cerebrovascular disease, and > 50% cerebral artery stenosis.

After CPB was initiated, the ascending aorta was cross-clamped and a cardioplegic solution was infused. Body temperature was measured at the nasopharynx and bladder, and was lowered to 28–32 °C. The α-stat strategy was applied for the pH management during CPB. FiO_2_ is initially set to 0.6 on the CBP oxygenator as a routine practice at our institution. After asystole was obtained and body temperature had stabilized, FiO_2_ was sequentially changed from 0.5 to 1.0, and back to 0.5, in the first half of the patients enrolled, and from 1.0 to 0.5, and back to 1.0, in the other half. Following a 5- to 10-min equilibration period for the three sequential FiO_2_ levels (T0–T2, respectively), blood gas analysis was performed using arterial and mixed venous blood sampled from the radial artery and venous reservoir of the CPB machine, respectively. A point-of-care analyzer (Gem®Premier™3000; Instrumentation Laboratory, Bedford, MA, USA) was utilized for the blood gas analysis. The pump flow rate of the CPB machine was recorded as the CO. Heart rate and mean blood pressure were also measured during the FiO_2_ changes.

### Protocol 2: off-pump coronary artery bypass grafting

This part of the study was a substudy of the CARROT trial, in which elective OPCAB patients were cluster-randomized on a monthly basis to receive FiO_2_ of either 0.3 or 0.8 during surgery. The length of postoperative hospital stay was the primary endpoint; other clinical outcomes will be compared in the CARROT trial. All participants taking part in the CARROT trial from November 1 to December 31, 2019 were consecutively enrolled in this substudy. Exclusion criteria for the CARROT trial included robot-assisted surgery, surgery via a thoracotomy, minimally invasive direct coronary artery bypass grafting, concomitant major surgery, any pulmonary condition requiring supplemental oxygen through any route before surgery, and preoperative use of mechanical circulatory assist devices.

After anesthesia was induced, the patients in the CARROT trial were mechanically ventilated with FiO_2_ of 0.3 or 0.8 during surgery based on the above-described cluster randomization (November 2019, FiO_2_ of 0.3; December 2019, FiO_2_ of 0.8). A pulmonary artery catheter (Swan-Ganz CCOmbo V 774HF75; Edwards Lifesciences, Irvine, CA, USA) was placed and connected to a continuous SvO_2_ and CO monitoring device (Vigilance II™; Edwards Lifesciences). The substudy protocol was performed during graft harvesting to ensure hemodynamic stability and minimal blood loss. FiO_2_ was changed from 0.3 to 0.8, and then back to 0.3, in patients allocated to receive FiO_2_ of 0.3 in the CARROT trial, while in those who received FiO_2_ of 0.8 it was changed from 0.8 to 0.3, and then back to 0.8 (T0–T2, respectively). FiO_2_ was held at each level for 5 to 10 min for stabilization, and blood gas analysis was performed at T0–T2 on arterial and mixed venous blood obtained from the radial and pulmonary arteries, respectively. No intravenous fluids were infused during the study protocol. Nasopharyngeal temperature, heart rate, and mean blood pressure were recorded during the FiO_2_ changes.

### Statistical analysis

The primary endpoint was the observed ΔSvO_2_ in response to a change in FiO_2_. Secondary endpoint was the difference between the observed and expected ΔSvO_2_ values (observed − expected ΔSvO_2_), i.e., the residual ΔSvO_2_.

Forty and 17 patients were recruited for protocol 1 (a pilot study) and protocol 2 (a substudy of the CARROT trial), respectively, without a sample size calculation. The statistical analysis was performed as follows. First, the observed ΔSvO_2_ was compared to zero (i.e., no change) using the one-sample *t*-test in on-pump cardiac and OPCAB patients. The observed ΔSvO_2_ of each patient was calculated as the absolute difference in SvO_2_ values measured at T0 versus T1, and T1 versus T2, thus giving two ΔSvO_2_ values per patient: the Bonferroni’s correction was applied. Second, we explored the distribution of the observed ΔSvO_2_ according to Hb concentration on a scatterplot, regardless of the surgery type. Assuming that the Hb concentration was constant during the change of FiO_2_ (T0–T2), the Hb concentration measured at T0 was taken as the representative value and used in the analysis. Pearson’s correlation analysis was performed to evaluate the association of Hb concentration with the observed ΔSvO_2_. Third, the observed ΔSvO_2_ was compared to the expected ΔSvO_2_ using a Bland–Altman plot, and the residual ΔSvO_2_ was calculated. The expected ΔSvO_2_ was calculated using Fick’s equation^7^

$${VO}_{2}=CO\times {(CaO}_{2}-{CvO}_{2})$$

where VO_2_ is oxygen consumption and CvO_2_ is the mixed venous oxygen content. As described earlier,

$${CaO}_{2}=\left({k}_{1}\times Hb\times {SaO}_{2}\right)+({k}_{2}\times {PaO}_{2})$$

and similarly,

$${CvO}_{2}=\left({k}_{1}\times Hb\times {SvO}_{2}\right)+({k}_{2}\times {PvO}_{2})$$

where PvO_2_ is the mixed venous oxygen partial pressure. Therefore,

$${VO}_{2}=CO\times \left\{\left({k}_{1}\times Hb\times {SaO}_{2}+{k}_{2}\times {PaO}_{2}\right)-\left({k}_{1}\times Hb\times {SvO}_{2}+{k}_{2}\times {PvO}_{2}\right)\right\}$$

We assumed that CO and VO_2_ remained constant from T0 to T2; hence, the following equation was established.

$$\left({k}_{1}\times Hb\times {SaO}_{2}[T0]+{k}_{2}\times {PaO}_{2}\left[T0\right]\right)-\left({k}_{1}\times Hb\times {SvO}_{2}\left[T0\right]+{k}_{2}\times {PvO}_{2}[T0]\right)$$

$$=\left({k}_{1}\times Hb\times {SaO}_{2}[T1]+{k}_{2}\times {PaO}_{2}[T1]\right)-\left({k}_{1}\times Hb\times {SvO}_{2}\left[T1\right]+{k}_{2}\times {PvO}_{2}\left[T1\right]\right)$$

or

$$\left({k}_{1}\times Hb\times {SaO}_{2}[T1]+{k}_{2}\times {PaO}_{2}\left[T1\right]\right)-\left({k}_{1}\times Hb\times {SvO}_{2}\left[T1\right]+{k}_{2}\times {PvO}_{2}[T1]\right)$$

$$=\left({k}_{1}\times Hb\times {SaO}_{2}[T2]+{k}_{2}\times {PaO}_{2}[T2]\right)-\left({k}_{1}\times Hb\times {SvO}_{2}\left[T2\right]+{k}_{2}\times {PvO}_{2}\left[T2\right]\right)$$

Rearranging this equation, the expected ΔSvO_2_ (T0–T1 and T1–T2) was calculated as follows:

$${The\,\, expected\,\, \Delta SvO}_{2}= \Delta {SaO}_{2}+\frac{{k}_{2}\times (\Delta {PaO}_{2}-\Delta {PvO}_{2})}{{k}_{1}\times Hb}$$

where ΔSaO_2_, ΔPaO_2_, and ΔPvO_2_ are the absolute difference of the SaO_2_, PaO_2_, and PvO_2_ values measured at T0 versus T1, and T1 versus T2. Fourth, an exploratory analysis was performed to identify factors potentially associated with the degree of ΔSvO_2_ according to ΔPaO_2_. Residual ΔSvO_2_ was compared among patients with and without chronic kidney disease, diabetes, hypertension, cerebrovascular disease, and congestive heart failure using the independent *t*-test or Wilcoxon rank-sum test after checking for normality. Only the residual SvO_2_ calculated at T0 versus T1 was used for this exploratory analysis. Fifth, we exploratively calculated the ΔHb equivalent that could increase DO_2_ to the same extent as ΔFiO_2_ of 0.5. For this calculation, it was assumed that CO remained unchanged, so the following equation was established. The ΔHb equivalent was calculated by rearranging the equation.

$${k}_{1}\times Hb \times {SaO}_{2}\left[{high\, \,FiO}_{2}\right]+{k}_{2}\times {PaO}_{2}[{high\, \,FiO}_{2}]$$

$$={k}_{1}\times \left(Hb +\Delta Hb \,\,equivalent\right)\times {SaO}_{2}[low\,\, {FiO}_{2}]+{k}_{2}\times {PaO}_{2}{[low \,\,FiO}_{2}]$$

All statistical analyses and data visualization were performed using R software (version 4.0.0; R Development Core Team, Vienna, Austria). Continuous variables are expressed as mean (SD) or median (IQR) as appropriate, and categorical variables are expressed as numbers (%). A P-value < 0.05 was considered significant.

## Supplementary Information

Supplementary Information.

## Data Availability

The data supporting this publication can be accessed by contacting the corresponding authors on reasonable request.
