# Supplementary material for: Effect of changes in inspired oxygen fraction on oxygen delivery during cardiac surgery: a substudy of the CARROT trial
Source: Sci Rep. 2021 Sep 9;11:17862. doi: 10.1038/s41598-021-97555-2 (PMC8429729; doi:10.1038/s41598-021-97555-2)
Supplement: Supplementary file 1 — Supplementary Information. [file 41598_2021_97555_MOESM1_ESM.docx]

**Effect of changes in inspired oxygen fraction on oxygen delivery during cardiac surgery: a substudy of the CARROT trial**

Karam Nam^1^, [Hye-Bin Kim](https://pubmed.ncbi.nlm.nih.gov/?term=Kim+HB&cauthor_id=24156702)^2^, [Young-Lan Kwak](https://pubmed.ncbi.nlm.nih.gov/?term=Kwak+YL&cauthor_id=27564562)^2^, Young Hyun Jeong^1^, Jae-Woo Ju^1^, Jinyoung Bae^1^, Seohee Lee^1^, Youn Joung Cho^2^, [Jae-Kwang Shim](https://pubmed.ncbi.nlm.nih.gov/?term=Shim+JK&cauthor_id=27564562)^2,*^, Yunseok Jeon^1,*^

^1^Department of Anesthesiology and Pain Medicine, Seoul National University Hospital, Seoul National University College of Medicine, Seoul 03080, Korea

^2^Department of Anesthesiology and Pain Medicine, Anesthesia and Pain Research Institute, Yonsei University College of Medicine, Seoul 03722, Korea

Karam Nam and [[Hye-Bin Kim](https://pubmed.ncbi.nlm.nih.gov/?term=Kim+HB&cauthor_id=24156702)](https://pubmed.ncbi.nlm.nih.gov/?term=Kim+HB&cauthor_id=24156702) contributed equally to this work.

^*^**Corresponding authors:** [Jae-Kwang Shim](https://pubmed.ncbi.nlm.nih.gov/?term=Shim+JK&cauthor_id=27564562) (aneshim@yuhs.ac) and Yunseok Jeon (jeonyunseok@gmail.com)

**Supplementary Table S1.** Hemodynamic data measured during the study protocols

|  | On-pump patients (n=36) | | | | | | Off-pump patients (n=17) | | | | | |
| --- | --- | --- | --- | --- | --- | --- | --- | --- | --- | --- | --- | --- |
|  | n=17 | | | n=19 | | | n=6 | | | n=11 | | |
|  | T0: 0.5 | T1: 1.0 | T2: 0.5 | T0: 1.0 | T1: 0.5 | T2: 1.0 | T0: 0.3 | T1: 0.8 | T2: 0.3 | T0: 0.8 | T1: 0.3 | T2: 0.8 |
| HR (beats/min) | NA | NA | NA | NA | NA | NA | 50 (47–69) | 50 (47–63) | 48 (45–59) | 64 (62–73) | 63 (59–75) | 63 (61–75) |
| MBP (mmHg) | 62 (6) | 65 (6) | 66 (4) | 61 (6) | 61 (9) | 67 (8) | 69 (67–74) | 67 (62–77) | 68 (63–69) | 73 (68–91) | 70 (64–89) | 71 (64–80) |
| CO (l/min) | 4.0 (0.4) | 3.7 (0.4) | 3.8 (0.5) | 4.2 (0.5) | 4.1 (0.6) | 3.9 (0.6) | 3.1 (2.9–3.3) | 3.1 (2.9–3.7) | 3.0 (3.0–3.6) | 3.5 (3.2–3.7) | 3.8 (2.9–4.1) | 3.6 (2.9–4.0) |
| CI (l/min/m^2^) | 2.5 (0.2) | 2.3 (0.2) | 2.4 (0.2) | 2.5 (0.2) | 2.4 (0.2) | 2.3 (0.2) | 1.9 (1.8–2.0) | 2.0 (1.8–2.1) | 1.9 (1.7–2.1) | 2.0 (2.0–2.0) | 2.1 (1.8–2.3) | 2.0 (1.8–2.3) |
| Hb (g/dl) | 7.5 (1.3) | 7.8 (1.0) | 7.6 (1.2) | 7.8 (1.4) | 8.1 (1.3) | 8.3 (1.4) | 11.6 (10.6–12.4) | 11.6 (9.9–12.7) | 11.3 (10.4–12.4) | 10.9 (10.0–12.4) | 10.5 (9.5–12.4) | 10.9 (10.1–12.3) |
| PaO_2_ (mmHg) | 266 (29) | 537 (51) | 262 (32) | 558 (61) | 263 (36) | 544 (74) | 89 (87–90) | 261 (244–278) | 110 (99–131) | 362 (290–394) | 130 (116–143) | 349 (322–368) |
| BT (°C)^*^ | 28.8 (1.4) | 29.9 (1.0) | 30.5 (1.1) | 29.5 (1.6) | 29.9 (1.5) | 30.1 (1.8) | 35.6 (35.4–35.9) | 35.6 (35.3–35.9) | 35.6 (35.3–35.7) | 36.1 (35.9–36.3) | 36.0 (35.9–36.4) | 35.9 (35.8–36.3) |

Data are expressed as mean (SD) or median (IQR). HR, heart rate; MBP, mean blood pressure; CO, cardiac output; CI, cardiac index; Hb, hemoglobin; BT, body temperature.

^*^Measured at the nasopharynx.
